# Relationship Between Identification of Functional Ankle Instability (IdFAI) Questionnaire Scores and Vertical Drop-Landing Kinetics in Netball Players: An Exploratory Study

**DOI:** 10.3390/jfmk11010027

**Published:** 2026-01-08

**Authors:** Darren-Lee Percy Kwong, Benita Olivier, Andrew Green

**Affiliations:** 1 Department of Sport and Movement Studies, Faculty of Health Sciences, University of Johannesburg, Johannesburg 2028, South Africa; andrewg@uj.ac.za; 2 Department of Sport, Health Sciences and Social Work, Faculty of Health and Life Sciences, Centre for Healthy Living Research, Oxford Institute of Applied Health Research, Oxford Brookes University, Oxford OX3 0BP, UK; bolivier@brookes.ac.uk

**Keywords:** functional ankle instability, dynamic postural stability, peak vertical ground reaction force, netball, multidirectional drop landing

## Abstract

**Background:** The Identification of Functional Ankle Instability (IdFAI) questionnaire is widely used to screen for functional ankle instability (FAI), but its link to objective landing kinetics in multidirectional sports like netball is not well-understood. This study aimed to (i) compare landing kinetics between idFAI stratified netball players, and (ii) examine associations between IdFAI scores with dynamic postural stability (DPS) indices and peak vertical ground reaction forces (PvGRF) during vertical drop landings. **Methods:** A cross-sectional exploratory study using a repeated-measures landing protocol was conducted on female university netball players (n = 24), stratified into FAI (n = 12) and non-FAI (n = 12) groups using the IdFAI (≥11 indicating possible FAI). Participants completed 18 unilateral drop jump landings in forward (FW), diagonal (DI), and lateral (LA) directions. Ground reaction forces (GRFs) were recorded to obtain DPS and PvGRF metrics (1000 Hz). Mann–Whitney U tests compared FAI groups, and Spearman correlations assessed associations (*p* < 0.05). **Results:** Players with FAI showed greater anteroposterior instability during LA landings (U = 33.5, *p* = 0.020, ES = 0.65). IdFAI scores correlated moderately with lateral anteroposterior deficits (rs = 0.473, *p* = 0.020, CI = 0.062–0.746). **Conclusions:** These findings suggest that players with greater FAI display increased anteroposterior instability during LA landings, with higher IdFAI scores moderately associated with these deficits. Despite the small exploratory, hypothesis-generating sample, the results emphasize the practical relevance of direction-targeted landing-stability training to improve DPS in vertical landings. This may provide insight into ankle-injury risk among FAI netball players, given that LA landings represent a documented ankle sprain mechanism.

## 1. Introduction

Functional ankle instability (FAI) is a prevalent condition marked by a recurrent sensation of instability or giving way in the ankle joint subsequent to a previous sprain [1,2,3]. Functional ankle instability impacts a significant number of individuals, particularly athletes, participating in high-impact activities and those with a history of ankle injuries [3]. Netball athletes are particularly prone to such symptoms due to the stop-start nature of the sport [4]. Specifically, injury prevalence among netball players is high, with 55–84% of netball players reporting their injuries, and up to 40% affecting the ankle [5]. Lateral jump landings are frequently associated with lateral ankle sprains (LAS), the most common injury in netball, with recurrence rates approaching 50% [6].

Landing kinetics describe ground reaction forces (GRFs) within the lower limb kinetic chain during ground contact. In FAI populations, GRF-derived measures help identify contributors to ankle instability during dynamic tasks [7,8]. Kinetic measures, including the Dynamic Postural Stability Index (DPSI), time to stabilization (TTS), and peak vertical GRF (PvGRF), are widely used to assess performance deficits and injury risk, typically through sagittal-facing drop landings of varying heights [9,10]. Conversely, landing direction has shown to influences dynamic postural stability (DPS) and PvGRFs, with deficits reported in healthy, post-injury, and FAI populations across lateral (LA), diagonal (DI), and forward (FW) landings [11,12,13]. This evidence may advocate for directionality as an important confounder in landing stability and GRF absorption ability. As netball is considered a high-intensity multidirectional sport, dynamic and reactive landing tasks of varying complexity (e.g., directionality) are essential to better reflect sport-specific demands [4].

Ankle instability is assessed using tools that capture symptoms, functional limits, and proprioceptive deficits [14]. The International Ankle Consortium recommends three self-reported instruments with established cutoff scores for evaluating subjective ankle instability: the Ankle Instability Instrument (AII), the Cumberland Ankle Instability Tool (CAIT), and the Identification of Functional Ankle Instability (IdFAI) [14,15]. Specifically, the IdFAI is a patient-reported outcome measure derived from the AII and CAIT [16,17], used to classify individuals with FAI. Its relative simplicity has contributed to its recent popularity and has facilitated validation across multiple languages [17,18,19,20]. Greater patient-reported instability often parallel quantifiable landing kinetic deficits, as both reflect underlying neuro-mechanical dysfunction affecting impact attenuation and stability. This link has been previously established in athletic and general populations [7,8,21].

Previous exploratory research linking self-reported FAI scores to landing kinetics has primarily utilized the validated CAIT questionnaire in relation to TTS measures of DPS. Higher CAIT scores have been associated with reduced anteroposterior and mediolateral stability during DI landings from 30 cm [21]. Conversely, no significant correlations between CAIT scores and TTS have been reported for FW drop landings (16 cm height) in general populations [22]. Differences across studies may arise from variations in sample selection, landing direction complexity, and drop-height intensity. More challenging tasks may be required to uncover deficits that are often concealed during simple FW-landing assessments.

As a relatively recent tool with less widespread use than the AII and CAIT, the IdFAI remains underexplored in relation to objective directional landing performance. While the CAIT provides a quick assessment of perceived ankle stability, it offers limited functional insight. In contrast, the IdFAI is more comprehensive, integrating perceived instability, mechanical deficits, and functional limitations, and its ease of use makes it particularly suitable for detailed evaluation in clinical and athletic populations [16]. Further comparative or cross-sectional research is needed to establish whether the IdFAI provides equivalent or complementary value in predicting landing-related outcomes in FAI populations, particularly its association with DPSI in netball.

This study therefore aimed to compare multidirectional kinetic measures (DPS and PvGRFs) between FAI and non-FAI groups, and to examine associations between IdFAI scores and these measures during FW, DI and LA drop landings. It was hypothesized that FAI participants would demonstrate higher DPS indices and PvGRFs than controls, and that IdFAI scores would positively correlate with these variables in a direction-specific manner.

## 2. Materials and Methods

### 2.1. Study Design

A cross-sectional, explorative study with a repeated-measures landing protocol was conducted to the following: (i) compare multidirectional drop-landing kinetics between netball players with and without functional ankle instability (FAI); (ii) examine associations between FAI scores and dynamic postural stability (DPS) indices, and between FAI scores and peak vertical ground reaction forces (PvGRF) during unilateral drop landings. A drop height of 40 cm was selected for single-leg drop protocols, as it reliably elicits measurable kinetic and kinematic responses while remaining safe for athletes [23], such as FAI-prone netball players. The choice prioritized reliability and participant safety rather than sport-specific landing heights, with multidirectional landing tasks accounting for netball-specific movement demands.

### 2.2. Ethical Approval

The study was conducted in accordance with the Declaration of Helsinki and approved by the University of Johannesburg Research Ethics Committee (approval number: [REC: 847-2020]). All participants provided written informed consent prior to participation.

### 2.3. Participants

A priori power analysis using G*Power 3.1.9.7 (f = 0.50, α = 0.05, rs = 0.70) indicated that 18 participants were required. Although the small sample limits statistical power, increasing the risk of Type II error and reducing generalizability, findings should be interpreted cautiously and as hypothesis-generating. The limited availability of sub-elite netball players, the intensive demands of biomechanical testing, and the exploratory aim of estimating preliminary effect sizes collectively supported the study’s feasibility, justifying the smaller cohort.

Twenty-four female athletes participating in university-level netball (age: 20.71 ± 2.14 years; height: 174.62 ± 9.21 cm; body mass: 67.1 ± 9.51 kg) met the required study criteria. Netball players were chosen due to their proficiency in jump landing and their heightened susceptibility to ankle injuries [5,6,24]. The IdFAI questionnaire was used to further stratify participants into FAI (n =12; age: 20.61 ± 2.09 years; height: 174.36 ± 4.72 cm; mass: 66.81 ± 6.51 kg) and non-FAI groups (n = 12; age: 20.84 ± 2.17 years; height: 173.76 ± 5.72 cm; mass: 68.12 ± 7.21 kg). The IdFAI cutoff (≥11) follows Simon et al.’s validation in the original development paper, where a receiver-operating characteristic (ROC)-derived score of ~10.3 was rounded to a practical threshold of 11 for identifying functional ankle instability [17].

Participants were eligible if they were 18–25 years old, actively engaged in a high-performance netball program for the past two years (~8 h/week, including strength and conditioning), and had played at least 60% (n = 8) of scheduled university cup fixtures, while remaining free of lower limb injury for ≥6 months. Exclusion criteria included prior lower limb surgery, diagnosed visual, vestibular, or neuromuscular impairments, and current or recent (within 6 months) use of ankle or knee braces. These criteria ensured a homogeneous sample with sufficient training and match exposure while minimizing factors affecting neuromuscular control, postural stability, and landing biomechanics. All participants provided written informed consent at least seven days before testing.

### 2.4. Procedures

Participants completed the idFAI questionnaire (Bloomington, IN, USA) to determine FAI, receiving a possible score out of 37 points per limb. The idFAI was used to obtain FAI scores for independent left and right limbs in all participants. Individuals were classified as having FAI if their score was ≥11, with increased severity represented by higher IdFAI scores [25]. As within-individual asymmetry (bias) was not the analytical focus, the most symptomatic limb (highest IdFAI score) was selected for analysis to maximize clinical relevance and sensitivity. Moreover, to align subjective severity with objective measures, consistent with FAI research practice [26]. Although limb dominance was not explicitly controlled for and may represent a potential confounder, 92% of the limbs included in the FAI analysis (n = 22 of 24) were dominant, minimizing the likelihood of dominance-related bias. Limb dominance was determined as previously described for single limb landings in a university-level netball cohort [27].

Stature and body mass were recorded to characterize baseline body composition but were not included in primary analyses. Stature was measured using a portable Seca 213 Stadiometer (Seca GmbH & Co. KG, Hamburg, Germany), and body mass was obtained from a 40 × 60 cm triaxial force plate (Bertec Corporation, Columbus, OH, USA) to the nearest 0.01 N and converted to kilograms.

**Figure 1:**
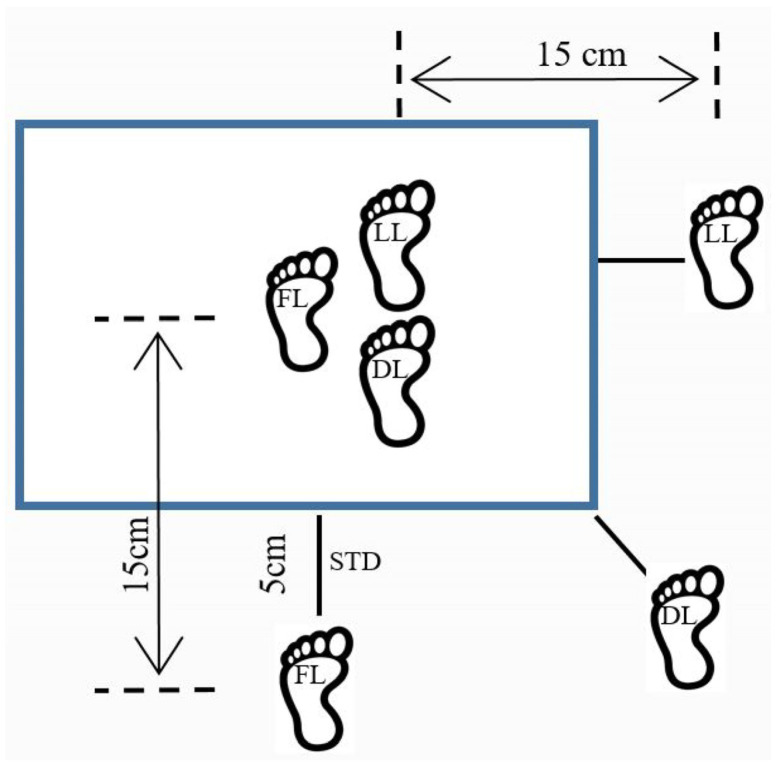
Exemplary multidirectional vertical drop-landing task setup for the left limb. The blue rectangle represents the force plate; FL = forward-directed landing (left limb); DL = diagonal-directed landing (left limb); LL = lateral-directed landing (left limb). The right limb was evaluated using an identical mirrored setup.

Participants completed a researcher-led warm-up consisting of 5 min of submaximal cycling at 60 W (Lode B.V., Groningen, The Netherlands), followed by dynamic lower-limb stretching. Vertical drop-landing trials were then performed, adapted from Huurnink [28] for height and direction. Each participant performed single-leg landings on the involved limb in FW, DI, and LA directions from a 40 cm plyometric box (HMS Fitness Equipment, Kraków, Poland) placed 5 cm from the force plate edge. Hands were placed on the iliac crests, and the contralateral limb was held off the ground with the knee in a comfortable, naturally flexed position (i.e., its angle set by the limb’s own muscle support rather than prescribed instruction). Participants focused on a visual target located three meters from the plate. Sticker-demarcated landing zones (±15 cm from the box) guided foot placement (Figure 1), and participants were instructed to stabilize within 10 s.

Participants performed two practice trials per landing direction before data collection. To minimize primary order effects such as fatigue, learning, or adaptation, block order was randomized within each limb, and testing alternated between limbs to evenly distribute fatigue. Three consecutive trials were collected per limb–direction combination (a single block) using a within-subject block randomization approach, followed by a randomized block of the alternate limb. The three trials were averaged to improve measurement reliability. Rest periods of 30 s between trials and two minutes between blocks were provided. Barefoot landings were used to minimize footwear-related confounds and enhance sensitivity to subtle variations in force distribution [29,30], controlling for between-individual footwear variation.

### 2.5. Data Analysis

All GRF data were collected using a Bertec FP 4060-05-PT at 1000 Hz and transferred to MATLAB, version R2023a ( MathWorks, Natick, MA, USA) for processing with custom scripts. The scripts served two purposes: (1) to calculate the dynamic postural stability index (DPSI) and its components: the anteroposterior (APSI), mediolateral (MLSI), and vertical (VSI) stability indices for the three landing directions; (2) to extract peak vertical ground reaction force (PvGRF), normalized to body weight and expressed as multiples of body weight (BWs). Computations for DPSI and its component indices were conducted as described by Wikstrom et al. [31]:

(1) APSI = √ [∑ (0 − x) 2/number of data points]

(2) MLSI = √ [∑ (0 − y) 2/number of data points]

(3) VSI = √ [∑ (body weight − z) 2/number of data points]

(4) DPSI = √ [∑ (0 − x) 2 + ∑ (0 − y) 2 + ∑ (body weight − z) 2/number of data points]

where x = the ground reaction force intercept from the sagittal force–time curve, y = the ground reaction force intercept from the frontal force–time curve and z = the ground reaction force intercept from the vertical force–time curve.

The DPSI was calculated from raw GRF data to preserve rapid fluctuations during landing that reflect true neuromuscular control, which would be attenuated by smoothing. Instead, using mean square deviations of the raw GRF enhances reliability by mathematically capturing brief accelerations and decelerations of the center of mass that contribute to postural stability [31].

For drop landings, the landing phase was defined from initial contact (IC), identified as vertical GRF > 10 N, and continued for 8 s. DPSI analysis was restricted to a 3 s window, as recommended by Wikstrom et al. [31], who reported strong correlations between DPSI values across 3–5 s (r = 0.96) and 3–10 s (r = 0.92). This interval best captures the short, explosive movements typical of team-sport performance.

### 2.6. Statistical Analysis

All statistical analyses were conducted in SPSS, version 30 (IBM, Chicago, IL, USA). Shapiro–Wilk tests indicated non-normal distribution for all dependent variables (*p* < 0.05); data are presented as median (IQR) and analyzed using nonparametric statistics. To address the first aim, Mann–Whitney U tests measures with Hodges-Lehmann median differences and 95% confidence intervals (CIs) were calculated to quantify group differences in IdFAI scores and landing kinetics between FAI and non-FAI groups (*p* ≤ 0.05). For effect size with non-parametric effect sizes (r) interpreted per Cohen [32]: 0.1–<0.3 small, 0.3–<0.5 moderate, >0.5 large. To address the second aim, Spearman’s rank-order correlations (rs) examined relationships between IdFAI scores and kinetic measures of DPS and PvGRFs across FW, DI, and LA landings (*p* < 0.05). Correlations were calculated for the complete sample and separately for questionnaire stratified FAI and non-FAI groups. To provide robust estimates of uncertainty, 95% confidence intervals CIs for each correlation coefficient were calculated using Fisher’s z-transformation, which accounts for sample size. Effect sizes were interpreted following Chan [33]: 0–0.2 none/poor, 0.3–0.49 fair, 0.5–0.69 moderate, 0.7–0.89 strong, and 0.9–1 very strong/perfect. Chan’s [33] thresholds of correlation strengths, developed for clinical and applied research [34], provide a more pragmatic framework than Cohen’s broader psychological-based guidelines. By offering finer distinctions within the moderate range (e.g., fair vs. strong), Chan’s system better captures the mid-range correlations typical of biomechanical data, where performance and injury outcomes arise from complex interactions among mechanical, neuromuscular, and physiological factors. This approach enables more meaningful interpretation of practically relevant associations that Cohen’s categories may underestimate.

Although multiple comparisons were performed, Bonferroni and Benjamini–Hochberg corrections were not applied due to the hypothesis-driven, exploratory nature of the study and the small sample (n = 24), where conservative corrections could increase Type II error [35,36]. Confidence intervals and effect size (r) were reported to strengthen interpretation given the limited sample size, while unadjusted *p*-values are presented and should be interpreted cautiously.

## 3. Results

Table 1 presents the FW, DI, and LA kinetic measures for the complete sample (n = 24), as well as for participants with FAI (n = 12) and without FAI (n = 12). The IdFAI scores, DPSI, APSI, MLSI, VSI, and PvGRF are reported as median (IQR) values for each group. Table 1 also provides the Mann–Whitney U test comparisons between the FAI and Non-FAI groups for all variables (IdFAI, DPSI, APSI, MLSI, VSI, and PvGRF).

### 3.1. Comparative IdFAI Scores, DPS Indices and PvGRFs Between Netball Players with and Without FAI

Mann–Whitney U tests were used to compare idFAI scores, DPSI, APSI, MLSI, VSI, and PvGRFs between FAI and non-FAI groups. As expected, the FAI group had significantly higher IdFAI scores than the non-FAI group (U = 2.5, *p* ≤ 0.001, r = 0.75), with a Hodges–Lehmann median difference of 13.84 units (95% CI 13.20–14.48).

A significant difference in APSI was observed during LA landings, with higher values in the FAI group. Mann–Whitney U analysis indicated a large effect (U = 33.5, *p* = 0.020, r = 0.65), with a Hodges–Lehmann median difference of 0.013 (95% CI 0.0097–0.0163), reflecting a meaningful group difference. No other significant differences were found for DPS or PvGRFs across groups (*p* > 0.05).

### 3.2. Associations of DPS Indices and PvGRFs with IdFAI Scores During Multidirectional Unilateral Drop Landings

Table 2 presents Spearman’s Rho correlation coefficients (rs) between GRF variables (DPSI, APSI, MLSI, VSI and PvGRFs) and IdFAI scores across the three groups: the complete sample (n = 24), FAI participants (n = 12), and non-FAI controls (n = 12). Metrics were assessed during directional drop landings in the FW, DI, and LA directions.

**Figure 2:**
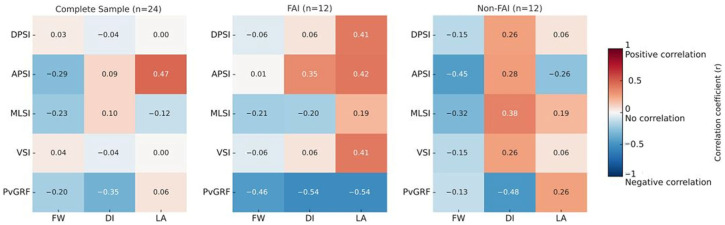
Heat map of Spearman’s correlations between IdFAI scores and landing variables (dynamic postural stability index—DPSI, anteroposterior stability index—APSI, mediolateral stability index—MLSI, vertical stability index—VSI, peak vertical ground reaction force—PvGRF) across forward (FW), diagonal (DI), and lateral (LA) drop landings. Darker blue shows stronger negative and darker red indicates stronger positive correlations. *p* < 0.05. Results shown for complete sample (n = 24), FAI (n = 12), and non-FAI (n = 12) groups.

Figure 2 expresses the Spearman correlations on a heat map. Correlation strength is represented by the color index scaled by correlation scores. Darker red indicates stronger positive associations, while darker blue indicates stronger negative associations. Conversely, lighter red and blue tones reflect weaker correlations.

A significant positive correlation was observed between higher IdFAI scores and greater APSI perturbations (rs = 0.473, *p* = 0.020), indicating a “fair” effect size according to Chan’s interpretation. Fisher’s z transformation calculating CI displayed considerable variability within the small sample (95% CI = 0.062–0.746). The finding is to be interpreted with caution. No other significant correlations were noted for FW, DI and LA landings for the composite sample or among FAI and non-FAI groups (*p* > 0.05). Insignificant correlations, spanning from poor to moderate, were observable for these correlations (Table 2).

## 4. Discussion

This study aimed to (i) compare DPSI, APSI, MLSI, VSI, and PvGRFs between FAI and non-FAI netball players, as well as (ii) examine associations between IdFAI scores and these kinetic measures during multidirectional unilateral vertical drop landings. It was hypothesized that FAI participants would demonstrate higher DPS indices and PvGRFs than controls, and that IdFAI scores would positively correlate with these variables in a direction-specific manner. The findings may modestly suggest that players with FAI may exhibit greater anteroposterior (AP) instability during LA landings, and that higher IdFAI scores are moderately associated with these deficits. Given the small, exploratory sample, results should be interpreted as hypothesis-generating rather than confirmatory and interpreted cautiously.

### 4.1. Sagittal Plane Instability and Lateral (LA) Landings

Well-established compensatory strategies in athletes with FAI may underlie the observed anteroposterior (AP) perturbations. Increased eccentric knee and hip extensor activation to attenuate landing forces can elevate sagittal plane moments [37], while greater pre-contact ankle dorsiflexion may stabilize the talocrural joint (i.e., talus locking) and limit frontal plane motion [13,38,39]. Together, these adaptations may shift force absorption proximally to the knee and hip, contributing to AP instability while minimizing frontal and vertical perturbations [39,40].

The positive correlation between higher IdFAI scores and greater AP instability suggests that increased FAI severity may drive reliance on enhanced talus locking, resulting in more proximal-dominant stabilization. The talus widens anteriorly, and forward movement of the ankle mortise during dorsiflexion enhances talar wedging, restricting frontal-plane motion while limiting sagittal-plane GRF absorption and ankle contribution. Since LA momentum–induced inversion is a key injury mechanism for lateral ankle sprain (LAS), the ankle may increase talus locking pre-IC to limit frontal-plane motion and prevent reinjury. However, due to the largely vertical nature of drop landings, reduced sagittal-plane contribution from the ankle shifts absorption demands to the knee and hip, requiring greater excursions to dissipate force. This mmay anifests as increased AP displacement rather than a coordinated ankle–knee–hip strategy (i.e., requiring less overall sagittal-plane proximal joint excursion). Consequently, worsening IdFAI symptoms may further amplify AP instability. Although these strategies may protect the unstable ankle, they could increase mechanical demands on the proximal joints. Such mechanisms are consistent with prior evidence of altered pre-contact foot positioning affecting proximal joint control in FAI and healthy athletes [7,41,42,43].

Given this, without supporting kinematic or electromyography (EMG) data, these interpretations remain speculative. The wide confidence interval (CI = 0.062–0.746) further indicates uncertainty in the strength of the association, likely due to the small sample size. Accordingly, these findings should be interpreted cautiously and regarded as preliminary and hypothesis-generating until replicated in larger, adequately powered cohorts.

### 4.2. Notable Non-Significant Correlations

Insignificant correlations were prevalent between IdFAI scores and landing kinetics across most directions (Table 2, *p* > 0.05). Notably a fair, though non-significant, positive correlation was observed between DPSI and IdFAI scores during LA landings in the FAI group (r = 0.411, *p* = 0.185), suggesting that greater self-reported instability may coincide with increased overall instability in frontal plane tasks, consistent with prior reports on the complexity of frontal plane momentum [38,41,42,44,45].

Unexpectedly, moderate negative correlations were observed between IdFAI scores and PvGRF during DI and LA landings (r = −0.544, *p* = 0.068). The previous literature suggests that individuals with FAI experience heightened PvGRFs due to limited proprioceptive feedback [46]. However, the inverse relationship here may indicate that those with greater FAI severity employ more efficient strategies to absorb PvGRFs, despite limited ankle contribution (i.e., increased talus locking). This supports the proposed shift toward greater proximal joint involvement mentioned previously [7,41,42]. Increased knee contributions leverage the quadriceps’ large eccentric force capacity, favorable lever mechanics, and substantial sagittal-plane range of motion to absorb PvGRF. However, this strategy may increase load on the knee and amplify AP instability during LA landings overtime. Nevertheless, these interpretations remain speculative and warrant further investigation through more comprehensive biomechanical techniques.

The predominance of non-significant correlations between DPS indices, PvGRF, and IdFAI scores should be interpreted cautiously. These results may reflect limited statistical power from small subgroup sizes (n = 12) rather than a true absence of association. Several fair-to-moderate correlations (Table 2) indicate potential biomechanical relationships that did not reach significance but may hold practical relevance. Therefore, these findings should be considered indicative, not conclusive, underscoring the need for studies with larger samples to confirm whether these patterns represent genuine biomechanical effects or sampling limitations.

Additionally, self-reported measures may not adequately reflect task-specific instability that manifests only under dynamic, high-demand conditions. Integrating objective biomechanical based assessments alongside subjective tools could therefore provide a more comprehensive understanding of FAI and its clinical applications.

### 4.3. Limitations

The limited associations observed between IdFAI scores and kinetic measures may partly reflect methodological constraints. The small sample size (n = 24) reduced statistical power and precision, increasing susceptibility to Type II errors and limiting generalizability. Although the findings offer valuable preliminary insights, they should be interpreted cautiously and verified in larger cohorts.

While kinetic analysis provides objective measures of DPS and PvGRF absorption, it offers limited insight into the specific joint and muscle contributions underlying these outcomes [47]. The absence of kinematic and EMG analyses constrains interpretation of movement quality, segment coordination, and neuromuscular strategies [48].

A key limitation is the limited ecological validity of the drop-landing tasks, including barefoot landings, non-sport-specific hand placement, absence of a ball or opponents, and the analysis of failed trials, all of which may influence landing biomechanics. Barefoot landings were used deliberately to reduce footwear-related confounds and increase sensitivity to subtle force variations [29,30]. While this enhanced detection of FAI-related differences, it limits transferability to in-game conditions. The authors acknowledge that barefoot testing limits ecological validity, as shoes can alter landing mechanics and strategies, cushioning, and subsequent force absorption. Therefore, findings may not fully generalize to in-shoe gameplay. Trials were repeated if the uninvolved limb contacted the plate or floor, if arms were not kept on the hips during stabilization, or if the participant missed the designated landing target. It is acknowledged that failed trials were not analyzed; future studies should consider including these, as most injuries occur during unsuccessful landings [6]. Assessment of failed trials may reflect injury risk from on-court falls. Although analyzing the affected limb introduces potential bias in this bilateral sport, the focus on between-group effects rather than within-individual asymmetry justifies this choice and likely does not affect primary outcomes.

Furthermore, ankle instability may not be fully captured by self-reported questionnaires such as the IdFAI [49]. Subjective reporting can be influenced by individual perceptions of instability and by clinical variability in diagnostic approaches [47,50], limiting the ability of self-reported tools to predict objective kinetic outcomes. Although the IdFAI is more comprehensive than the CAIT in simultaneously capturing mechanical deficits and functional limitations [16], it does not fully assess mechanical instability, a key component of ankle instability. This may limit its ability to inform kinetic deficits in DPS arising from mechanical limitations.

Lastly, multiple comparisons increase the risk of false positives. Given the exploratory nature of this study, *p*-values were not adjusted for multiple comparisons to minimize the risk of Type II errors and avoid overlooking potentially meaningful relationships. However, this approach increases the likelihood of Type I errors, and thus, the observed associations should be interpreted cautiously and considered preliminary and hypothesis-generating until replicated in larger, confirmatory studies.

### 4.4. Implications for Netball Practice

Ankle injuries are common in netball, accounting for ~37.5–40% of all injuries [5], with LA jump landings associated with the highest incidence of ankle sprains [6]. Up to 50% of players may experience recurrent, often bilateral, sprains following an initial injury [51], particularly in athletes with FAI deficits. The idFAI questionnaire, although modest, aligns with previous findings indicating heightened risk in FAI limbs during lateral landings [6].

Practically, reduced AP stability during LA landings may imply an increase in knee joint stress by limiting frontal plane ankle motion and subsequent energy absorption. This may be characterized in FAI limbs with excessive pre-contact dorsiflexion [38]. Repeated LA landings could therefore impose cumulative knee loading, highlighting the importance of eccentric strength and endurance in the quadriceps and gluteus maximus for effective proximal force absorption [52]. Recent studies highlight the importance of multidirectional landings, particularly diagonal landings, in netball [27]. Repeated exposure to varied landing patterns has shown to improve proprioception, neuromuscular control, and movement competency in youth netball players [53]. Such exposure could potentially lead to improved DPS responses during landing, promoting effective PvGRF absorption.

Accordingly, plyometric training programs incorporating multidirectional landings are therefore recommended. In addition, eccentric-focused conditioning of the lower limb extensors may further reduce injury risk and improve performance in athletes with FAI. This can be achieved through direction-specific depth jumping and landing exercises. These drills utilize the stretch-shortening cycle, with muscles and tendons eccentrically absorbing force on landing, followed by rapid concentric contraction to generate maximal force.

### 4.5. Future Studies

Recruiting large samples of elite netball players remains challenging due to small team sizes (≈15 players), underscoring the need for strategic sample size planning. Future studies focusing on collaborative, multi-provincial recruitment during interprovincial tournaments may improve participant representation and enhance statistical robustness. Furthermore, larger studies seeking to confirm finding in the current study should focus on multidirectional netball landing should integrate kinetics with kinematic and EMG analyses to more comprehensively identify mechanisms of suboptimal landing biomechanics and injury risk.

To enhance ecological validity, continuing research should incorporate sport-specific footwear, netball-relevant landing tasks, and reactive, game-like scenarios to improve the translational relevance of findings to on-court performance and injury risk. Notably, failed trials should be considered an important metric for landing analysis in prospective studies, having relevance to injury etiology. To adequately investigate self-reported methods, integrated objective biomechanical assessments alongside subjective tools could provide a more comprehensive understanding of FAI and its impact on performance.

## 5. Conclusions

These findings suggest that players with greater FAI display increased anteroposterior instability during lateral landings, with higher IdFAI scores moderately associated with these deficits. These exploratory findings, limited by sample size and task-specific constraints, should be interpreted as hypothesis-generating. They may potentially indicate eccentrically targeted plyometric training interventions to reduce potential proximal joint injury risk in a netball cohort. Future research should recruit larger, diverse netball cohorts and employ multidirectional, sport-specific landing assessments that combine biomechanics and subjective measures to improve netball ecological validity and elucidate injury mechanisms.

## Figures and Tables

**Table 1:** Descriptive statistics and group comparisons for the Identification of Functional Ankle Instability (IdFAI) questionnaire scores and kinetic measures across landing directions.

		Complete Sample	FAI	Non-FAI	Mann–Whitney U Test (FAI vs. Non-FAI)		
		(n = 24)	(n = 12)	(n = 12)	*p* (Exact)	Mean Difference (Hodges–Lehmann)	95% CI
IdFAI Score	-	10.50 (13.5)	18.42 (6.52)	4.58 (1.29)	≤0.001 *	13.84	(13.20/14.48)
DPSI	FW	1.12 (0.24)	1.14 (0.24)	1.12 (0.31)	0.630	0.020	(−0.202/0.242)
	DI	1.20 (0.25)	1.21 (0.23)	1.17 (0.64)	0.551	0.040	(−0.345/0.425)
	LA	1.18 (0.28)	1.10 (0.28)	1.20 (0.25)	0.410	−0.100	(−0.312/0.112)
APSI	FW	0.068 (0.02)	0.064 (0.05)	0.069 (0.02)	0.347	−0.005	(−0.035/0.025)
	DI	0.064 (0.03)	0.061 (0.03)	0.070 (0.03)	0.755	−0.009	(−0.033/0.015)
	LA	0.048 (0.01)	0.051 (0.02)	0.038 (0.02)	0.020 *	0.013	(0.009/0.016)
MLSI	FW	0.054 (0.03)	0.055 (0.05)	0.061 (0.03)	0.478	−0.006	(−0.039/0.027)
	DI	0.070 (0.02)	0.071 (0.02)	0.069 (0.04)	0.799	0.002	(−0.023/0.027)
	LA	0.080 (0.02)	0.077 (0.03)	0.083 (0.04)	0.291	−0.006	(−0.034/0.022)
VSI	FW	1.12 (0.24)	1.13 (0.25)	1.12 (0.31)	0.590	0.010	(−0.215/0.235)
	DI	1.19 (0.25)	1.21 (0.23)	1.16 (0.64)	0.551	0.050	(−0.335/0.435)
	LA	1.18 (0.28)	1.10 (0.28)	1.20 (0.25)	0.410	−0.100	(−0.312/0.112)
PvGRF (BWs)	FW	3.92 (0.24)	3.96 (0.56)	3.44 (0.93)	0.843	0.520	(−0.094/1.134)
	DI	3.79 (0.96)	3.67 (0.93)	3.97 (0.94)	0.590	−0.300	(−1.048/0.448)
	LA	3.66 (0.93)	3.68 (0.99)	3.41 (0.82)	0.443	0.270	(−0.457/0.997)

FW—forward; DI—diagonal; LA—lateral; APSI—anteroposterior stability index; MLSI—mediolateral stability index; VSI—vertical stability index; DPSI—dynamic postural stability index; PvGRF—peak vertical ground reaction force; idFAI score—Identification of Functional Ankle Instability questionnaire score; BWs—multiples of body weight; *—Statistically significant difference at *p* < 0.05.

**Table 2:** Spearman’s correlation coefficients (rs) between DPS indices and PvGRFs against IdFAI scores in multidirectional vertical drop landings.

	FW			DI			LA		
	*r_s_*	*p*	95% CI	*r_s_*	*p*	95% CI	*r_s_*	*p*	95% CI
Complete sample (n = 24)									
DPSI	0.033	0.878	(−0.375/0.431)	−0.045	0.834	(−0.441/0.356)	0.003	0.989	(−0.401/0.406)
APSI	−0.286	0.176	(−0.623/0.141)	0.094	0.662	(−0.322/0.480)	0.473 *	0.020	(0.062/0.746)
MLSI	−0.234	0.271	(−0.586/0.192)	0.102	0.634	(−0.315/0.487)	−0.121	0.574	(−0.501/0.299)
VSI	0.043	0.843	(−0.367/0.439)	−0.045	0.834	(−0.441/0.356)	0.003	0.989	(−0.401/0.406)
PvGRF (BWs)	−0.195	0.360	(−0.586/0.192)	−0.352	0.092	(−0.586/0.192)	0.059	0.785	(−0.586/0.192)
FAI (n = 12)									
DPSI	−0.060	0.854	(−0.613/0.533)	0.060	0.854	(−0.533/0.613)	0.411	0.185	(−0.239/0.806)
APSI	0.007	0.983	(−0.569/0.579)	0.354	0.258	(−0.294/0.779)	0.416	0.178	(−0.233/0.809)
MLSI	−0.207	0.519	(−0.702/0.422)	−0.204	0.526	(−0.700/0.425)	0.193	0.548	(−0.433/0.694)
VSI	−0.060	0.854	(−0.613/0.533)	0.060	0.854	(−0.533/0.613)	0.411	0.185	(−0.239/0.806)
PvGRF (BWs)	−0.456	0.136	(−0.827/0.192)	−0.544	0.068	(−0.864/0.090)	−0.544	0.068	(−0.864/0.090)
Non-FAI (n = 12)									
DPSI	−0.153	0.634	(−0.671/0.464)	0.256	0.422	(−0.382/0.728)	0.058	0.857	(−0.534/0.612)
APSI	−0.453	0.139	(−0.826/0.195)	0.285	0.369	(−0.357/0.744)	−0.263	0.409	(−0.732/0.376)
MLSI	−0.321	0.308	(−0.763/0.325)	0.380	0.223	(−0.270/0.792)	0.190	0.554	(−0.436/0.692)
VSI	−0.153	0.634	(−0.671/0.464)	0.256	0.422	(−0.382/0.728)	0.058	0.857	(−0.534/0.612)
PvGRF (BWs)	−0.132	0.684	(−0.658/0.481)	−0.482	0.122	(−0.839/0.163)	0.256	0.422	(−0.382/0.728)

FW—forward; DI—diagonal; LA—lateral; *r_s_*—Spearman’s correlation coefficient; *p*—*p*-Value; DPSI—dynamic postural stability index; APSI—anteroposterior stability index; MLSI—mediolateral stability index; VSI—vertical stability index; PvGRF—peak vertical ground reaction force; FAI—functional ankle instability; BWs—multiples of body weight; *—Statistically significant correlation at *p* < 0.05.

## Data Availability

The original contributions presented in this study are included in the article. Further inquiries can be directed to the corresponding author.

## Abbreviations

The following abbreviations are used in this manuscript:

IdFAI	Identification of Functional Ankle Instability Questionnaire
FAI	Functional Ankle Instability
AP	Anteroposterior
ML	Mediolateral
DPS	Dynamic Postural Stability
DPSI	Dynamic Postural Stability Index
APSI	Anterior–Posterior Stability Index
MLSI	Medial-Lateral stability index
VSI	Vertical Stability Index
PvGRF	Peak Vertical Ground Reaction Force
BWs	Multiple of Body Weight
FW	Forward
DI	Diagonal
LA	Lateral
